# *The unfunded priorities:* an evaluation of priority setting for noncommunicable disease control in Uganda

**DOI:** 10.1186/s12992-018-0324-2

**Published:** 2018-02-20

**Authors:** Beverley M. Essue, Lydia Kapiriri

**Affiliations:** 1 University of Sydney, Sydney, NSW 2006 Australia; 2 McMaster University, 1280 Main Street W, Hamilton, ON L8S 4K1 Canada

**Keywords:** Noncommunicable diseases, Priority setting, Uganda, Evaluation, Mixed-methods

## Abstract

**Background:**

The double burden of infectious diseases coupled with noncommunicable diseases poses unique challenges for priority setting and for achieving equitable action to address the major causes of disease burden in health systems already impacted by limited resources. Noncommunicable disease control is an important global health and development priority. However, there are challenges for translating this global priority into local priorities and action. The aim of this study was to evaluate the influence of national, sub-national and global factors on priority setting for noncommunicable disease control in Uganda and examine the extent to which priority setting was successful.

**Methods:**

A mixed methods design that used the Kapiriri & Martin framework for evaluating priority setting in low income countries. The evaluation period was 2005–2015. Data collection included a document review (policy documents (*n* = 19); meeting minutes (*n* = 28)), media analysis (*n* = 114) and stakeholder interviews (*n* = 9). Data were analysed according to the Kapiriri & Martin (2010) framework.

**Results:**

Priority setting for noncommunicable diseases was not entirely fair nor successful. While there were explicit processes that incorporated relevant criteria, evidence and wide stakeholder involvement, these criteria were not used systematically or consistently in the contemplation of noncommunicable diseases. There were insufficient resources for noncommunicable diseases, despite being a priority area. There were weaknesses in the priority setting institutions, and insufficient mechanisms to ensure accountability for decision-making. Priority setting was influenced by the priorities of major stakeholders (i.e. development assistance partners) which were not always aligned with national priorities. There were major delays in the implementation of noncommunicable disease-related priorities and in many cases, a failure to implement.

**Conclusions:**

This evaluation revealed the challenges that low income countries are grappling with in prioritizing noncommunicable diseases in the context of a double disease burden with limited resources. Strengthening local capacity for priority setting would help to support the development of sustainable and implementable noncommunicable disease-related priorities. Global support (i.e. aid) to low income countries for noncommunicable diseases must also catch up to align with NCDs as a global health priority.

**Electronic supplementary material:**

The online version of this article (10.1186/s12992-018-0324-2) contains supplementary material, which is available to authorized users.

## Background

As in most other low income countries (LICs), noncommunicable diseases (NCDs) have become a major cause of mortality and morbidity in Uganda due to an epidemiological shift that has resulted from demographic and nutritional transitions in the population. Low income countries are now faced with a double disease burden from infectious diseases and NCDs [1] and this poses a unique challenge for priority setting in health systems that are already resource strapped.

The growing burden of NCDs in LICs threatens to undermine economic and social development [2]. Addressing this burden has become a global health and development priority, culminating in a 2011 United Nations High Level meeting on the prevention and control of NCDs [3]. Global commitment is reinforced in the Sustainable Development Goals, which include a target focused on reducing pre-mature mortality and morbidity from NCDs [4]. This strong international direction has resulted in policy initiatives in many countries.

However, many barriers still remain to achieving global NCD targets [5]. The East Africa NCD Alliance Post-2015 Initiative identified several barriers that were stalling local action to control NCDs, including: an absence of specific local targets and indicators in National plans; misalignment between development assistance partners’ (DAPs) priorities and country priorities, weak implementation frameworks, weak health system capacity to manage chronic conditions and weak monitoring and surveillance infrastructure [6].

While some studies have examined the resource requirements to enhance NCD control in Uganda [7], less is understood about how the resources should be allocated, the potential trade-offs in the health system and the challenge posed by the double burden of disease in this context. Previous studies have sought to understand national level prioritization processes in Uganda [8, 9]. However, most of these have focused generally on the national level health system and very few have focused on specific conditions [10–12]. Evaluating priority setting for NCDs presents an interesting and critical case of how LICs are grappling with addressing the double disease burden with limited resources as well as how global priorities have been translated into local priorities and action.

Kapiriri and Martin devised a conceptual framework [13] that has been validated for evaluating priority setting in low and middle income countries [14]. Table 1 defines the key parameters for evaluating successful priority setting, the evidence that is required for its evaluation (i.e. the means of verification (MOVs)), and the objectively verifiable indicator(s) (OVI). This framework provides a standardised approach for the systematic evaluation of priority setting (Table 1).

**Table 1:** Parameters for evaluating priority setting with corresponding indicators and means of verification

	Parameters of successful priority setting	Objectively Verifiable Indicators (OVI)	Means of Verification (MOV)
Contextual Factors	Conducive political, economic, social and cultural context	Relevant contextual factors that may impact priority setting	Follow up intermittent interviews with local stakeholders, systematic longitudinal observations, relevant reports, media reports.
Pre-requisites	Political will	Degree to which the politicians support the set priorities	Follow up intermittent interviews with local stakeholders, systematic longitudinal observations, relevant reports, media reports.
	Resources	Budgetary and human resource allocation to the health sector	National budget documents
	Legitimate and credible institutions	Degree to which the priority setting institutions can set priorities, public confidence in the institution	Stakeholder and public interviews
	Incentives	Material and financial incentives	National budget documents
The priority setting process	Stakeholder participation	Number of stakeholders participating, number of opportunities each stakeholder gets to express opinions	Observation at meetings/minutes of meetings, media reports, special reports
	Use of clear priority setting process/tool/methods	Documented priority setting process and/or use of priority setting framework	Observation at meetings/minutes of meetings, media reports, special reports
	Use of explicit relevant priority setting criteria	Documented/articulated criteria	Observation at meetings/minutes of meetings, media reports, special reports
	Use of evidence	Number of times available data is resourced/number of studies commissioned/existing strategies to collect relevant data	Observation at meetings/minutes of meetings, media reports, special reports
	Reflection of public values	Number and type of members from the general public represented, how they are selected, number of times they get to express their opinion, proportion of decisions reflecting public values, documented strategy to enlist public values, number of studies commissioned to elicit public values	Observation at meetings/minutes of meetings, study reports, meeting minutes and strategic plans
	Publicity of priorities and criteria	Number of times decisions and rationales appear in public documents	Media reports
	Functional mechanisms for appealing the decision	Number of decisions appealed, number of decisions revised	Observations at meetings/minutes at [of] meetings, media reports, special reports
	Functional mechanisms for enforcement	Number of cases of failure to adhere to priority-setting process reported	Observation at meetings/minutes of meetings, media reports, special reports
	Efficiency of the priority setting process	Proportion of meeting time spent on priority setting, number of decisions made on time	Observation at meetings/minutes of meetings, annual budget documents, health system reports
	Decreased dissentions	Number of complaints from stakeholders	Meeting minutes, media reports
	Allocation of resources according to priorities	Degree of alignment of resource allocation and agreed upon priorities, times budget is re-allocated from less prioritized to high prioritized areas, stakeholder satisfaction with the decisions	Annual budget reports, evaluation documents
	Decreased resource wastage/misallocation	Proportion of budget unused	Budget documents, evaluation reports
	*Improved internal accountability/reduced corruption*	Number of publicized resource allocation decisions	Evaluation reports, stakeholder interviews, media reports
	*Increased stakeholder understanding, satisfaction and compliance with the priority setting process*	Number of stakeholders attending meetings, number of complaints from stakeholders, percentage of stakeholders that can articulate the concepts used in priority setting and appreciate the need for priority setting	Observation at meetings/minutes of meetings, special reports, stakeholder satisfaction survey, media reports, stakeholder interviews, evaluation reports
Implementation of the set priorities	*Improved internal accountability/reduced corruption*	Number of publicized resource allocation decisions	Evaluation reports, stakeholder interviews, media reports
	*Strengthening of the priority setting institution*	Indicators relating to increased efficiency, use of data, quality of decisions and appropriate resource allocation, percentage of stakeholders with the capacity to set priorities	Training reports, evaluation reports, budget documents
	*Impact on institutional goals and objectives*	Percentage of institutional objectives met that are attributed to the priority setting process	Evaluation reports, special studies
Priority setting outcomes	*Impact on health policy and practice*	Changes in health policy to reflect identified priorities	Policy documents
	*Achievement of health system goals -improved population health -reduction in health inequalities -fair financial contribution -responsive health care system*	Percent reduction in DALYs, percent reduction of the gap between the lower and upper quintiles, percentage of poor populations spending more than 50% of their income on health care, percentage of users who report satisfaction with the healthcare system	Ministry of Health documents, Demographic and Health Surveys, commissioned studies
	*Improved financial and political accountability*	Number of publicized financial resource allocation decisions, number of corruption instances reported, percentage of the public reporting satisfaction with the process	Reports, media reports, interviews with stakeholders
	*Increased investment in the health sector and strengthening of the health care system*	Proportion increase in the health budget, proportion increase in the retention of health workers, percentage of the public reporting satisfaction with the health care system	National budget allocation documents

Key: Non-italics = immediate parameters (i.e. can be assessed in a budget cycle); Italics = delayed parameters (i.e. assessed over a longer term, such as the planning cycle)

This study aimed to use the Kapiriri & Martin (2010) conceptual framework to evaluate the influence of national, sub-national and global factors on priority setting for NCD control in Uganda and examine the extent to which priority setting was successful.

## Methods

### The analytical framework

This evaluation was conducted using the Kapiriri and Martin framework (Table 1). In this study, priority setting was defined as the processes through which interventions are ordered and resources allocated between competing programs and diseases. The focus was on human and financial resources.

### Study design

This evaluation used mixed methods. Four categories of data sources were used as MOVs to evaluate the parameters for successful priority setting. Table 2 summarizes which methods were used to evaluate each of the parameters in this evaluation. The focus was on national priority setting and the influence of global, national and subnational factors.

**Table 2:**
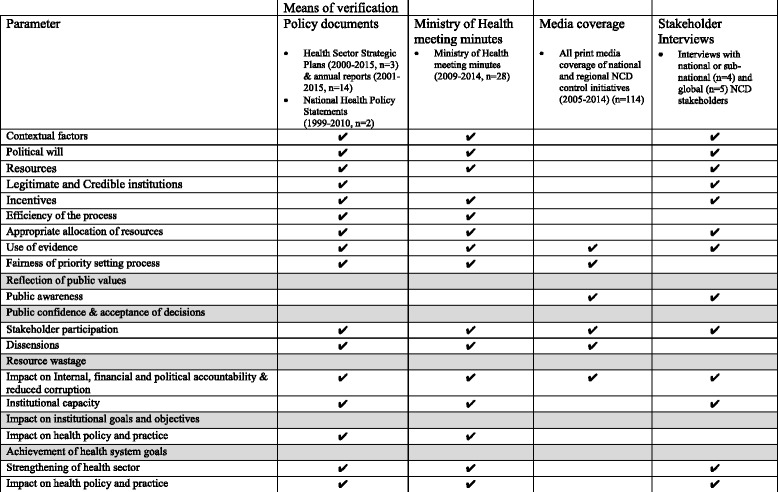
Summary of the data sources used as means of verification to evaluate each priority setting parameter

Shading indicates the parameters that could not be assessed in this evaluation

### Data sources

#### Document review

This included policy documents and meeting minutes.

The policy documents outlined the strategic focus and implementation of health initiatives in Uganda, with a focus on those relevant to the control of NCDs. The documents covered the period 2000–2015.

The minutes of the Ministry of Health senior management meetings documented the participants attending those meetings and key policy areas discussed. They provided information about whether clear and fair priority setting processes were followed. The meeting minutes were from 2009 to 2015.

#### Media analysis

Media reports reflected public discourse on and public opinion of policies to control NCDs. A search was run in the FACTIVA print media database to identify articles from the following sources, printed between January 1st 2005 and December 31st, 2015: Daily Monitor, New Vision and The Observer. These sources collectively have the widest readership. Inclusion criteria were articles that referred to NCDs, and more specifically, the following conditions: cardiovascular diseases, chronic obstructive pulmonary disease, cancers and diabetes. These four conditions were selected to align with the focus of the global agenda on addressing NCDs [3].

#### Key informant interviews

The interviews used pilot-tested open-ended questions and explored the following topics: priority setting processes in Uganda, including for NCDs, the importance of NCDs as a priority compared to other areas, the criteria used to prioritize, stakeholder engagement in priority setting and the publicity of priority setting decisions and criteria (see Additional file 1). Interviews were conducted by telephone or face to face by the principal investigator (LK) and trained Ugandan research assistants. Interviews lasted approximately 45 minutes and were audio recorded with permission from the respondents.

Interviews were conducted between 2013 and 2015 with global, national, and sub-national stakeholders who were involved in influencing NCD policy. The interviews conducted in 2013 provided a baseline; and the follow up interviews allowed for evaluation of issues affecting the implementation of priority areas. The study team, in collaboration with local partners at the Ministry of Health, compiled a preliminary list of the main policy makers, development assistance partners (DAP) and other key individuals involved in NCD control in Uganda. These individuals were invited to participate in interviews for this study. Snowball sampling was used to recruit additional stakeholders. This recruitment strategy entails a process where existing participants identify potential other participants from their network of colleagues or acquaintances who would have a relevant perspective to contribute to the study.

### Data analysis

The Kapiriri & Martin Framework was used as the overarching framework for the analysis. The data sources were reviewed and critically assessed to distil evidence and extract examples on the OVI for each parameter. A data collection form was developed based the OVIs for each parameter (Table 1).

For the document review and media analysis, one researcher carried out the analysis of the policy documents (BE), Ministry of Health meeting minutes (BE) and media reports (LK) in close consultation with the study team. The data collection form was used to identify and catelogue examples of documented OVIs. The absence of information relevant to a particular parameter was also noted.

Interviews were transcribed verbatim by a third party service and the validity of the transcription was verified against the audio recording by a research assistant. Transcripts were read in their entirety and the data collection form was used to identify and categorise examples of OVIs in the interview data.

Each data source was first analysed separately and then all data from each source were triangulated across each parameter from the framework to assess the degree to which priority setting was successful.

The study received ethics approvals. All participants provided written informed consent.

## Results

### Summary of the data:

There were 47 documents included in this study: two National Health Policy statements, three Health Sector Strategic Plans (HSSP), 14 annual reports that corresponded to each year of the strategic plans and minutes from 28 Ministry of Health senior management meetings (Table 2). All documents were available in the public domain, except the minutes, which were provided by the Ministry of Health. The media search identified 114 news articles published in the print media on the topic of NCDs, cardiovascular disease, chronic obstructive pulmonary disease, cancers or diabetes. Interviews were conducted with 9 stakeholders from government, DAPs or research organisations. Stakeholders were involved in NCD control at either the national (*n* = 4) or the global level (*n* = 5).

### Evaluation of priority setting for NCDs

Table 3 provides a summary of the key findings for the parameters that could be evaluated (17/22). The following parameters were not evaluated due to the unavailability of data specified by their corresponding MOVs: reflection of public values, public confidence in and acceptance of decisions, resource wastage, institutional goals and objectives and achievement of health system goals.

**Table 3:** Summary of evaluation findings for each parameter of the Kapiriri & Martin framework

	Parameters of successful priority setting	Key findings
Contextual factors	Conducive political, economic, social and cultural context	Contextual factors had both positive (e.g. UN High level meeting) and negative (e.g. staff turnover in the MOH) influences on the process
Pre-requisites	Political will	Political will documented in the policies, but inadequate resources to support successful PS from the outset. Ministry of Health seen as having a legitimate and credible role to set priorities. No evidence of incentives to set priorities for NCDs
	Resources	
	Legitimate and Credible institutions	
	Incentives	
The Priority setting process	Successful process	
	Stakeholder participation	Wide stakeholder involvement but major players (e.g. DAPs) were able to exert influence on the process and the selection of priorities
	Use of clear priority setting process/tool/methods	The NHP and HSSP provided the framework for priority setting and defined the process.
	Use of explicit relevant priority setting criteria	Consistency among stakeholders in the criteria identified as being most relevant for establishing national health priorities but lack of a defined process to systematically assess all relevant criteria.
	Use of evidence	There was a commitment to evidence-informed priority-setting demonstrated by the use of existing data in the process, the identification of data gaps and priority given to addressing the data gaps.
	Reflection of public values	–
	Publicity of priorities and criteria	The decisions about what interventions to prioritize and the criteria used to make these decisions were not publicized
	Functional mechanisms for appeal and enforcement of the decision	There were no reported appeals. There were no documented mechanisms to ensure adherence to the conditions of a fair process
	Efficiency of the priority setting process	The quality of decisions was improving but the probability of implementing the identified NCD objectives did not improve
	Dissentions	Calls for increased funding and more equitable funding for NCDs in the media.
	Public understanding and confidence in the process	–
	Allocation of resources according to priorities	NCDs were identified as a priority area in the NHPs and HSSPs, but there was an ongoing challenge of insufficient resources allocated to support policy and program development
	Decreased resource wastage/misallocation	–
	*Increased stakeholder understanding, satisfaction and compliance with the Priority setting process*	Stakeholders had a good understanding of the process and were somewhat satisfied though recognised that the process was not fully transparent. There was no evidence that stakeholders failed to comply with decisions.
Implementation of the set priorities	*Impact on internal, financial and political accountability and corruption*	Greater internal, financial and political accountability were still needed to minimize opportunities for corruption and mismanagement to interfere with the process.
	*Strengthening of the priority setting institution*	Malalignment between priorities and resource allocation and lack of transparency for the allocation of resources and implementation of priority areas indicates that there is scope for further strengthening of the PS institutions
	*Impact on institutional goals and objectives*	–
Priority setting outcomes	*Impact on health policy and practice*	Increase in health policies to support NCD control and some impact on practice.
	*Achievement of health system goals* *-improved population health* *-reduction in health inequalities* *-fair financial contribution* *-responsive health care system*	–
	*Increased investment in the health sector and strengthening of the health care system*	Evidence of increased investment and a commitment to strengthen the health care system to address NCDs

Non-italics = immediate parameters; Italics = delayed parameters

- = unable to assess. For *Achievement of health system goals,* − = too early to assess

*NCD* noncommunicable diseases, *PS* Priority setting

### Conducive political, economic, social and cultural context

For this parameter, evidence was assessed on the political environment (i.e. whether priority setting could be participatory and fair), the economic context (i.e. whether there were health financing arrangements in place to support priority setting and implementation) and the social and cultural context (i.e. whether priorities and priority setting were considered acceptable and feasible).

While the 2011 UN High Level meeting was central to progressing the global agenda to address NCD control by establishing a global commitment [3] and galvanizing momentum, all of the stakeholders reported that Uganda may not have had sufficient technical expertise to prioritize NCD control and progress this agenda locally. One global stakeholder commented:

*“there was a high level agreement to do more and do something about NCDs…countries then went back and discussed this and maybe started a process of domestic consultation about priorities and developed their own national level action plans…But it’s taken that long and there was a sort of pent-up demand for technical support…”(G03).*

In relation to health financing globally and domestically to support LICs to prioritise addressing NCDs, the same global stakeholder commented:

*“financing the implementation of NCD action plans is a huge issue and problem…there isn’t so far any sort of attempt to set up a Global Fund for NCDs… if you look at the total envelope of developmental assistance [for NCDs], it’s an extremely small percentage of current levels of development assistance…the main form of financing NCDs have come more from national or domestic sources which is a big struggle for a very low income country... this is a big headache about how to pay for this [NCDs] because they’ve got so many other health priorities and their own resources for raising money domestically are very modest.” (G03).*

There was evidence assessed that the momentum to address health priority areas, including NCDs also suffered from the challenge of a high turnover in government. One stakeholder remarked: “*who is with you today may not be with you next week*” (N01). This meant that NCDs often lacked a strong and consistent champion. This issue was reiterated in follow-up interviews as a key barrier to the implementation of NCD priorities.

Other influential factors identified by stakeholders included the lack of coherent messaging about what needed to be done to improve NCD control at a global level and stigma associated with NCDs as they continued to be perceived as lifestyle disorders (G02).

### Prerequisites for priority setting

The framework identified four pre-requisites to successful priority setting namely; political will, legitimate and credible priority setting institutions, availability of resources and incentives.

There was evidence of some political vision regarding the need to address NCD control in the National Health Policies [15, 16], which documented that Uganda had already entered an epidemiological transition. However, there was a lack of evidence in the HSSP annual reviews of progress or action in line with this political vision. Most of the national stakeholders felt that strong and consistent political will and leadership to address NCDs had been lacking and this was linked to the point made above about turnover of staff in the Ministry of Health. For example:

*“Political leadership changed … [s/he] had started to make positive changes then [s/he] is taken away…then we spent time when there is no successive Minister, the ones who are there are not sure of their stance, so I think there is total disruption of the political leadership [for NCDs]”(N05).*

All stakeholders indicated that the Ministry of Health had a legitimate and credible role as the main institution responsible for national level priority setting for health and this was supported by the documented processes in the HSSPs and Ministry of Health meeting minutes. The Ministry of Health established a designated office for NCDs in 2006 which convened a technical working group that was responsible for compiling evidence to support priority setting for NCDs. All national stakeholders perceived this working group as having a legitimate role in informing priority setting but acknowledged that the Office of NCDs was severely under-resourced.

The health system lacked resources to address NCDs. Specific resource challenges discussed by stakeholders and noted in the annual reports included: access to medicines, human resource capacity, specialist training and late release of funds. One national stakeholder remarked that, at the time of the interviews in 2015, there was still less than 70% of human resources in place to support the NCD-related priorities (N02). A lack of resources for NCDs was also cited as an explanation for failure to achieve NCD-related targets in all of the annual reports that covered the period of 2005–2015 [17–27].

There were no explicit incentives in place to progress the NCD control agenda at the national level.

### Priority setting processes

According this the framework, the processes through which priorities are set should fulfil the following criteria: a) be participatory; b) be based on clear and explicit processes; c) be evidence based; d) ebased on explicit relevant criteria; e) have mechanisms for publicizing the rationales for the decisions, appealing and revising the decisions, and enforcing decisions; f) allocation of resources should align with the set priorities; g) have buy-in and support from stakeholders [13, 28]. This section is organized according to these parameters.

#### Stakeholder participation:

There was evidence, in the policy documents and the Ministry of Health minutes, of wide stakeholder involvement. For example, a range of clinicians, bureaucrats and representatives from civil society, including various national and global health organisations contributed to priority setting in the following ways: attended the joint review mission meetings (where the HSSP reports are developed), attended the Ministry of Health senior management meetings, made submissions to the meetings, or were consulted as experts.

Other stakeholders who were considered to have legitimate participation included representatives from civil society. Organisations such as the International Diabetes Foundation, the East African NCD Alliance and the Ugandan NCD Alliance were influential in coalescing civil society around NCDs and helped to raise the profile of NCDs in Uganda. Mental Health Uganda and Uganda Schizophrenia were also identified as strong champions at all levels of priority setting for mental illness.

Besides the involvement of civil society organisations, there was little mention of the engagement of patient representatives or the general public in the priority setting processes.

There were mixed perceptions about the legitimacy of DAPs. For example, NCDs were identified as a global health priority by the WHO General Assembly and as a result, the Ministry of Health was then responsible for aligning national priorities with global priorities set by the WHO. This was seen as important for progressing national efforts to get NCDs on the national policy agenda.

However, the influence and involvement of other DAPs was less well accepted. This was captured by a comment from one national stakeholder who indicated that while the Health Policy Advisory Committee had ultimate decision making power over which areas were identified as priorities, various DAPs had influential roles on that committee and this affected the transparency of decision-making (N03).

DAPs also influenced resource allocation and implementation. Stakeholders discussed tensions between areas being prioritized because of DAP interest rather than using a systematic assessment of more objective criteria such as the disease burden and the availability of effective interventions. For instance, polio continued to attract substantial funding in Uganda despite its decreasing prevalence and incidence. One national stakeholder commented:

*“[DAPs] are playing a big role because they dictate where their money is going… We had suggested much earlier that they should let us set our priorities and they fund our priorities but that has not worked because they have their own interests… So, unfortunately they are still playing a very big role”(N03).*

A key issue raised by global and national stakeholders was the need for better coordination of DAPs and DAP funding. It was common for several DAPs to be working in the country in the same health area (e.g. cervical cancer) and this was viewed as inefficient. For example, one global stakeholder commented:

*“There probably needs to be coordination, because within country there may be 5 or 6 NGOs [working in cervical cancer] …And the Ministry of Health and … sometimes in some countries there’s too many partners and players, and a coordination role is required”(G05).*

#### Use of clear priority setting processes, tool or method and explicit relevant criteria:

Review of the policy framework documents indicated that priority setting for NCDs fell under the remit of the health sector planning process. The National Health Policy and the HSSPs provided the framework for priority setting in the health sector and the annual reports documented progress in addressing priority areas. The process tended to include the following steps: the NHP and HSSPs set out the framework for identifying all potential health priority areas; the Ministry of Health used policy to govern and set standards; and districts implemented priorities.

There was consistency among stakeholders in the criteria identified as being most relevant for establishing national health priorities., including: prevalence, burden of disease, consequences of disease; costs; cost-effectiveness of available interventions, and impacts on the economy and other sectors. Burden of disease and cost-effectiveness data were viewed as essential criteria though it was recognised that locally-derived data were not routinely available or used.

Stakeholders commented that the selection of priorities tended to happen more implicitly because there was not a defined process for systematically assessing all of the relevant criteria. As a result, other factors were able to weigh in and influence which priority areas were ultimately identified and implemented. For NCDs, the global health agenda was seen as an influential factor. For example, one national stakeholder summarised:

*“that’s what guides the priorities [in Uganda] so to speak…whatever they adopt there [globally] we’re supposed to customize in this context and advise the Ministry in line with their[global] priorities (N02)”.*

Another stakeholder discussed the potential for the global agenda to eventually shift the focus of DAP assistance and local priorities in the future, for example:

*“the current debate about the shape and form of the SDGs is so important because that sets out the blueprint for the next fifteen years and the donors will follow and that’s where the money will go and then the countries will follow it… it’ll have a very direct effect on what are the priorities of countries.” (G03).*

The WHO list of best buys was discussed as a global resource that LICs should use as criteria for selecting which health programs to prioritize for implementation. But there was a recognition that the best buys were not necessarily relevant in all contexts and this could detract from the implementation of other important programs, including some relevant to NCD control. For example, one global stakeholder commented:

*“There are a set of identified “best buys” and plenty of countries [including Uganda]… have said “this is not a very big priority for us in our country because the rate of alcohol use in our country is very low, so doing an advertising ban for alcohol doesn’t make good sense”, for example… other interventions that were not on the “best buys” list might and indeed do constitute important interventions for countries” (G01).*

#### Use of evidence:

WHO projections of the impact of non-action were commonly cited in the policy documents to make the case for needed action on NCD control [20–27].

The annual reviews from 2007 onwards cited an increase in health service use, morbidity and mortality associated with some NCDs, including: COPD, CVD and cancers in Uganda [20–27]. This evidence was used to justify the need for a NCD risk factor baseline survey to provide policy makers with better data on the prevalence of\NCD risk factors and the potential magnitude of the burden that could be caused by inaction on NCDs.

There was an acknowledgement in HSSP annual reports from 2003 to 2010 that a lack of evidence on the burden of NCDs and risk factors for NCDs had led to inaction [18–21, 29–32]. The following challenge was noted explicitly in several annual reports: “the lack of data [on NCDs] is often taken for non-existence of the problem and limited attention has thus far been given to the problem of NCDs” [17, 18, 33].

National stakeholders reiterated the challenges that Uganda faced for developing evidence-informed priorities. For example:

*“globally they are there, but locally we did not have that information… about the prevalence of these diseases [NCDs] and what we need to do about it… people with the money understand numbers… so if you say “Oh it’s a big problem”, but how big? [This evidence] will help in the advocacy for what to do about which NCD’s” (N04).*

Stakeholders indicated a need for evidence on effective local interventions as there was a recognition that research from other settings, especially high income countries, was not directly transferrable. For example:

*“I think we still lack research evidence on the most appropriate interventions for NCDs in our setting… There might be interventions that work in developed countries, but in a limited resource setting, we need to identify the most appropriate that we can afford to implement in our setting”.*

#### Publicity of decisions:

All of the policy documents were available online however, the extent to which these documents were accessible to the public is unclear.

The media publicized some events such as the start of the risk factor survey, health campaigns linked to NCD-related risk factors, the establishment of new cancer and CVD treatment centres and enhanced support for existing health care facilities. But stakeholders felt that the publicity of NCD priorities and programming was generally inadequate, for example:

*“I don’t hear anything happening about NCDs in the media … you never hear that there is a workshop of the NCD programme” (N05).*

The media coverage of NCDs did not include information about what decisions were made, what interventions to prioritize and the criteria used to make these decisions.

#### Mechanisms for appeal, revisions and enforcement:

The policy documents did not explicitly outline the mechanisms for appeal, revision or enforcement of decisions and there were no instances of documented appeals or revisions in the data.

#### Efficiency of the process:

For this parameter data were evaluated to determine evidence of returns on the resources (in terms of time) invested in setting priorities vis a vis their implementation.

There was evidence in the policy documents and minutes that priority setting for NCDs was becoming more efficient over time. For example, the HSSPII indicated four NCD-related target indicators for the period of 2005/06 to 2010/11: 1) obtain national baseline data on NCD risk factors; 2) produce national NCD policy, standards and guidelines; 3) develop national programme for NCD prevention and control and, 4) achieve 30% increase in health facilities with functional integrated NCD clinics. While there was no progress noted in achieving any of these targets in the 2005/2006 annual report, progress towards the first target was reported in 2007/08 and progress towards the first three targets was reported in the 2010/11 annual report. At the Ministry of Health meetings, discussion of NCDs progressed from identifying them as an emerging problem (2009) to greater attention devoted in the meetings to reviewing proposals for NCD-related programs (3 meetings in 2011) and discussion of progress on specific indicators such as the implementation of the NCD risk factor survey and health worker trainings (2014 meetings).

Evidence of implementation delays and gaps for example, the 3 year delay in implementing the NCD risk factor survey suggested that there were still inefficiencies in priority setting for NCDs.

#### Allocation of resources according to priorities:

While NCDs were included as a priority health condition in the Uganda National Minimum Health Care Packages since 2001, the package had a legacy of insufficient funding. For example, in 2002 it was funded at US$7 per capita instead of the required US$28 [34]; and in 2011, while the estimated cost was US$48, the 2015 per capita health expenditure was still less than US$12 [35]. For NCDs in particular, in 2014–2015, only 34.7% of the budget allocated for the control of communicable diseases and NCDs was availed [35].

In addition to the lack of resources for implementation in the health sector more generally, but especially for “lower priority” programs such as NCDs, two challenges were discussed by stakeholders. First the funded, or higher prioritized areas, were often those that were the focus of existing DAP action, as discussed above. Second, in many cases, resources were already linked to existing priority areas, for example, achieving the MDGs. Stakeholders commented that this created challenges for making new investments in the health sector for emerging areas. In a typical comment about misalignment between the stated priorities in the policy documents and the funding allocated to NCDs, one global respondent stated:

*“I think that is the main reason there hasn’t been a dramatic increase in resources …they’ve (donors) already got their commitments up to 2015 in the form of the MDGs…, the battleground is happening now about the presence… of NCDs in the SDG framework and what that might lead to” (G03).*

#### Stakeholder understanding, satisfaction and compliance:

Most (8/9) of the stakeholders interviewed had knowledge of how priorities were set in Uganda and were somewhat satisfied with the processes, but some (3/9) felt there could be greater transparency, particularly on the choice of priority health areas.

An issue raised by some stakeholders was that the policies tended to set out ambitious objectives which could not be adequately resourced or implemented. For example:

*“So, even when we prioritize things, they remain in the books and are not funded. So, that discourages people to prioritize.” (N03).*

There was no evidence that stakeholders failed to comply with decisions.

#### Dissensions:

There were calls to address NCDs in the media, often in response to cases of high profile deaths from NCDs, which suggested that there was some dissatisfaction with the inaction or delays in the progress of enacting priorities.

A key area that received media attention was the economic impact of NCD treatment on patients. The media reports highlighted the significant costs of medications for example, as much as half of a household’s monthly income for one vial of insulin to treat diabetes. There were calls to prioritize action on implementing equitable funding for medicines in order to address NCD-related catastrophic health expenditure [36].

### Implementation of set priorities

There was evidence of the implementation of NCD-related priorities, for example: setting up the Office of NCDs within the Ministry of Health; development of a national NCD policy and strategy; implementation of the risk factor survey; media awareness campaigns and training of health providers.

However, as discussed above, some stakeholders felt that some of the priorities were unrealistic and never fully implemented. For example:

*“They never implement fully…the plan has more big expectations than can be realized that’s how it is” (N01).*

#### Internal, financial and political accountability:

Lack of accountability was identified as an issue by many stakeholders who felt decisions were not communicated well within and outside of the Ministry of Health.

Stakeholders felt that there was scope for improvements in internal financial accountability as concerns were raised about how funding had been used. They discussed instances where funding allocated to NCD control, such as for the NCD survey, were not actually available which delayed the implementation of the survey (N04). In another example, one stakeholder commented:

*“…because of lack of transparency … the director can decide to prioritize and if your activity does not fall in the priority of the head of the department you may end up with nothing and they use that money… by the time you go to check there is no money in your account and you are told you have to wait for money for the next quarter. The next quarter comes still again it is inadequate, so now it’s two quarters…only one activity has been funded” (N05).*

There were indications that efforts were being made to improve accountability and transparency in the funding of NCD policies. For example, a decision was documented in the 2014 Ministry of Health meeting minutes that required costing estimates to be submitted with all new Bills to ensure that the cost implications were considered and on the public record.

Corruption in the health sector was identified as an issue by two stakeholders that impacted the availability of funds for priority areas. A formal presentation was minuted in a 2010 meeting on the need to address corruption and make explicit initiatives to combat corruption in the HSSPIII and second National Health Policy.

#### Strengthening of the priority setting institution:

Where there was evidence of improvements in the parameters relating to the use of evidence and the quality of decision making, as discussed above, which suggested that there had been progress in strengthening the priority setting institutions. However, the malalignment between priorities and resource allocation and lack of transparency for the allocation of resources and implementation of priority areas indicated that there was scope for further strengthening of the priority setting institutions.

### Priority setting outcomes

The following parameters were evaluated to determine whether outcomes of priority setting were achieved: impact on health policy and practice; and increased investment in the health system.

#### Health policy and practice:

From 2011, there was a clear transition in the policy momentum to support NCD control, with more detail provided in the policy documents and minutes on specific bills and acts in development by Parliament. Despite this, few were actually funded or implemented due to insufficient resources.

#### Investment in and strengthening of the health sector:

There was evidence of increased investment in the health sector, some of which was allocated to addressing NCDs. For example, funding from the International Diabetes Foundation supported in part, the Ministry of Health’s Office of NCDs and later funded the risk factor survey.

Opportunities were identified to further strengthen the health sector’s capacity to address NCDs. For example, by harnessing existing infrastructure used for other infectious diseases (e.g. HIV). One national stakeholder commented:

*“HIV [providers] have realised that they are presenting the HIV treatment but their clients are dying of various diseases. So, they are finding a way of including NCDs into their programs” (N03).*

Mental health provided an example where strong leadership fostered an environment that supported ongoing research, advocacy, policy and service development and eventually ear-marked funding for additional mental health services.

## Discussion

The Kapiriri & Martin framework was used to evaluate priority setting for NCDs in Uganda. To the authors’ knowledge, this is the first systematic evaluation of the impact of global, national and sub-national factors on priority-setting for NCDs in a LIC. This evaluation makes an important contribution to the literature by providing an assessment of how priorities for NCD control – which have largely been generated and driven by global stakeholders – have been translated and adopted within the priority setting processes in a LIC.

There was evidence that NCDs were prioritized and new health policies were developed to support NCD control; a finding that is consistent with a recent analysis of the landscape of NCD initiatives in Uganda [37]. This may be a reflection of the growing global interest in and support for NCDs. International declarations and priorities often translate into national priorities and tend to be championed by DAPs who provide the resources to support the implementation of the priorities [38]. However, NCDs seem unique in that while they have garnered global interest and commitment, the resource commitment from both government and DAPs has remained inadequate. In 2012, more than 70% of bi-lateral donor assistance in Uganda was still allocated to infectious diseases, with the majority going to HIV/AIDs [8]. It is hence not surprising to have found in this evaluation that insufficient resources were thought to explain the malalignment between NCDs as priorities and the actual resource allocation and implementation in the health system.

If countries are to be successful in meeting NCD targets there is an urgent need for DAP aid to catch up and better align with and support NCDs as a global priority area [39, 40]. Moreover, there is a need for greater political leadership and accountability to ensure that NCD related priorities are adequately supported and implemented in national planning processes. The trend in multilateral donor assistance in Uganda is showing promising signs of change – there has been an increase in support for health system strengthening, still just over 30% of all funding in 2011/2012 [8]. But if used appropriately, this has the potential to help strengthen the health system to better address NCDs and the double disease burden in the long term.

The results highlight challenges for priority setting in the context of a changing disease burden. Much of the data that has been collected on the burden of disease in Uganda and used in priority setting is old and may not reflect the current epidemiological profile [41]. The pivot in global health priorities to include NCDs has meant that many countries, including Uganda, have been faced with the challenge of setting priorities for their health system without sufficient local evidence of the magnitude of the NCD problem and evidence on the effectiveness of potential solutions in their setting [7]. This has limited the ability of the health system to be responsive to emerging issues such as NCDs.

This evaluation found the lack of local epidemiological data was a factor that contributed to some complacency in priority setting, inadvertently framing NCDs as a non-issue and as such, a lower priority compared to infectious diseases. While there has certainly been global leadership and advice on NCD priorities (e.g. from the Global Burden of Disease studies) and interventions to support NCD control, for example, from the Disease Control Priorities studies [42] and the WHO Best Buys [43] these have not been universally adopted, in part due to a lack of evidence of their local effectiveness and appropriateness [29, 40]. Ongoing investment in research on NCDs in LICs should help to fill the evidence gaps and support evidence-informed, locally relevant priority setting for NCD control [2, 31].

Investment in new disease areas (i.e. NCDs) may require some disinvestment from existing areas that require ongoing support (i.e. HIV/AIDS, TB and malaria control). While there are frameworks to guide resource allocation (e.g. PBMA, A4R, MCDA), they tend to be applied in higher-income countries and few have been shown to be effective in supporting disinvestment decision making in LICs in practice [32]. This evaluation highlighted the need for guidance for LICs, above and beyond cost-effectiveness research, on how to make these decisions in the face of competing resource requirements, particularly those brought on by the demands of a double disease burden.

This study has limitations. While the sample size for interviews seems small, it included all the key stakeholders involved in priority setting for NCDs in Uganda. The media analysis only included print media. It is possible that television and radio coverage of NCDs may have had different emphasis. The following parameters were not evaluated due to the unavailability of data: reflection of public values, public confidence in and acceptance of decisions, resource wastage, impact on institutional goals and objectives and, achievement of health system goals. A public awareness survey or interviews with the public would help to ascertain the public values about priority setting and the assigned priority areas. This is relevant for priority setting decisions with major resource implications (e.g. new technologies) and to ensure that issues that are of importance to the public are accounted for (e.g. the high cost of NCD medicines for patients). In addition, a comprehensive assessment of budget allocations was not carried out as we could not access to all budget documents. Where possible, the evaluation draws on budget information available in the public domain [35]. These MOVs could have provided additional insights into the success of priority setting for NCDs.

## Conclusion

This evaluation revealed the challenges that Uganda faced in prioritizing NCDs in the context of a double disease burden with limited resources and constrained health system capacity. While priority setting for NCDs was somewhat successful, this evaluation highlighted the need to further strengthen the national priority setting institutions in Uganda to support the development of sustainable and implementable NCD priorities. In addition, to address the implementation gap, global support (i.e. aid) to low income countries for NCDs must catch up to align with NCDs as a global health priority.

The findings from this evaluation provide important context for future evaluations of Uganda’s progress towards achieving the global NCD control 25 × 25 target, which specifies 25 voluntary NCD related targets for countries to achieve by 2025. With NCDs now firmly on the policy agenda in Uganda, effective priority setting will be critical for meeting the burden caused by NCDs in this population as well as in other LICs.

## Additional file

Additional file 1: Interview guide (DOCX 36 kb)

## Abbreviations

DAP: Development Assistance Partner
HSSP: Health Sector Strategic Plan
LIC: Low income country
MDG: Millennium Development Goals
MOV: Means of Verification
NCD: Noncommunicable diseases
NHP: National Health Policy
OVI: Objectively Verifiable Indicators
SDG: Sustainable Development Goals
UHC: Universal Health Coverage
UN: United Nations
WHO: World Health Organisation
