# Supplementary material for: The unfunded priorities: an evaluation of priority setting for noncommunicable disease control in Uganda
Source: Global Health. 2018 Feb 20;14:22. doi: 10.1186/s12992-018-0324-2 (PMC5819649; doi:10.1186/s12992-018-0324-2)
Supplement: Supplementary file 1 — Interview guide (DOCX 36 kb) [file 12992_2018_324_MOESM1_ESM.docx]

I**nterview topics discussed in Stakeholder interviews**

1. Priority setting processes in Uganda, including for NCDs.
2. The importance of NCDs as a priority relative to other areas.
3. The criteria used to prioritize in the health sector.
4. The ways in which stakeholders are engaged in priority setting.
5. The publicity of priority setting decisions and criteria.
